# Bioactive Oxadiazoles 3.0

**DOI:** 10.3390/ijms25116027

**Published:** 2024-05-30

**Authors:** Antonio Palumbo Piccionello

**Affiliations:** Department of Biological, Chemical and Pharmaceutical Sciences and Technologies (STEBICEF), University of Palermo, Viale delle Scienze, Ed. 17, 90128 Palermo, Italy; antonio.palumbopiccionello@unipa.it

Heterocycles are fundamental moieties for the construction of new compounds with perspective applications ranging from drugs to materials [1,2,3,4].

In this wide panorama we focused our attention to the sub-class of Oxadiazoles. Oxadiazoles are electron-poor five-membered aromatic heterocycles that contain one oxygen and two nitrogen atoms. The oxadiazoles, namely 1,2,3-, 1,2,4-, 1,2,5-, and 1,3,4-regioisomers, together with *N*-oxides, benzo-fused, and non-aromatic derivatives, present a wide application range from material science to explosives and bioactive compounds [5,6,7,8]. In the latter field, there are many possibilities, and oxadiazoles have been revealed to be active as antitumoral agents [5,9], neuroprotective compounds [10], antimicrobials [8,11], among others.

Some bioactive oxadiazoles have reached the market or are in the advanced clinical trials stage, such as oxolamine, ataluren, raltegravir, capeserod, azilsartan, furazabol, sydnophen, zibotentan, opicabone, etc. Moreover, the oxadiazole skeleton is also present in some natural compounds, such as phidianidine and quisqualic acid, providing new inspiration for the synthesis of bio-inspired drugs. Another important issue is the use of oxadiazoles in medicinal chemistry as amide and ester isosters and as peptido-mimetics, offering a well-established tool for drug design. All of these aspects have increased interest in these heterocycles, increasing the impact of oxadiazoles in the field of bioactive compounds. This Special Issue is the continuation of our previous Special Issues “Bioactive Oxadiazoles” [12] and “Bioactive Oxadiazoles 2.0” [13] and is intended to offer a wide panorama of the potential applications of these compounds toward all diseases.

The research fields explored in this Special Issue include the discovery of new compounds with various biological activities such as: antibacterial 1,2,4-oxadiazole derivatives [14], antiplasmodial 1,2,5-oxadiazoles [15], 1,2,4-oxadiazole derivatives as nematicides [16] and 1,3,4-oxadiazoles with anticancer activity [17]. Moreover, the synthetic accessibility of bioactive 1,2,4-oxadiazoles at room temperature, was reviewed [18]. These researches are just some examples of the fascinating and expanding field of oxadiazoles’ chemistry, that represent an emerging field with growing potential.
